# A case-aware feature modulation framework for defect classification in power lines

**DOI:** 10.1038/s41598-026-53001-9

**Published:** 2026-05-27

**Authors:** Nyan Farooq Ezzulddin, Zainab Mohammed Abdulkareem, Saif Mohamed Baraa Alsabti, Raid Gaib, Ali M. Elhenidy

**Affiliations:** 1 Electronic and control, Engineering Technical College-Kirkuk, Northern Technical University, Mosul, Iraq; 2 Technical College of Engineering, Al-Bayan University, Baghdad, Iraq; 3 Computer Engineering and Control Systems Department, Faculty of Engineering, Mansoura University, Mansoura, Egypt

**Keywords:** High-voltage insulators, Contamination classification, Engineering, Mathematics and computing

## Abstract

Conductors and grounded transmission towers are separated by non-conductive overhead transmission line insulators are known as materials. They frequently meet with problems once they are put into use mechanical or electrical pressure and environmental pollution. It is important to carry out regular inspections to avoid power failures because adverse working conditions may lead to insulation breakdown. To do this, this study proposes a new method of classifying high-voltage surface conditions of insulators are given depending on the picture, which is founded on deep convolutional neural networks (CNNs). We suggest MS-CADFM-SSL, a new multi-task model of defect classification of various components of power lines. The strategy incorporates a communal EfficientNet Multi-scale case-dependent dynamic feature modulation, orthogonality regularization, backbone with multi-scale case-dependent dynamic feature modulation and self-supervised pretraining to learn jointly generalized representations and keep task specific discriminative features. The framework was tested on five nonhomogeneous defect cases has strong performance with regard to precision, recall, F1-score, and accuracy. Industrially, it guarantees dependable identification of severe errors, decreases computing expenses, and real time checking of the transmission lines. In comparison to traditional single-task or naive multitask.MS-CADFM-SSL has better adaptability to visually diverse and imbalanced datasets, where focus areas are physically meaningful due to Grad-CAM visualizations. Despite difficulties using infrequent or delicate anomalies and depending on fixed images, the structure offers a scalable basis of automated defect test. Future extensions consist of multimodal and temporal integration of data, semi-supervised learning and predictive maintenance prioritization to improve dependability and workability.

## Introduction

Overhead transmission lines (OHTLs) are required in order to transmit and distribute power. OHTLs are used to carry electrical energy to load centers where power-generating sources are located. The operation of the power transmission system has always been based historically on power plants, OHTLs, and substations; most of these buildings were installed decades ago and are now approaching or even exceeding their designed service life^1^. Such degrading infrastructure leads to such problems as corrosion and material degradation, which increases the cost of maintenance and may even jeopardize the safety and reliability of the system. Despite the challenges, monitoring of conditions and maintenance of the system are essential in ensuring dependability of the systems in OHTL. Governments and power corporations should take initiatives to prevent power failures and ensure that there is always adequate electricity supply.

**Fig. 1:**
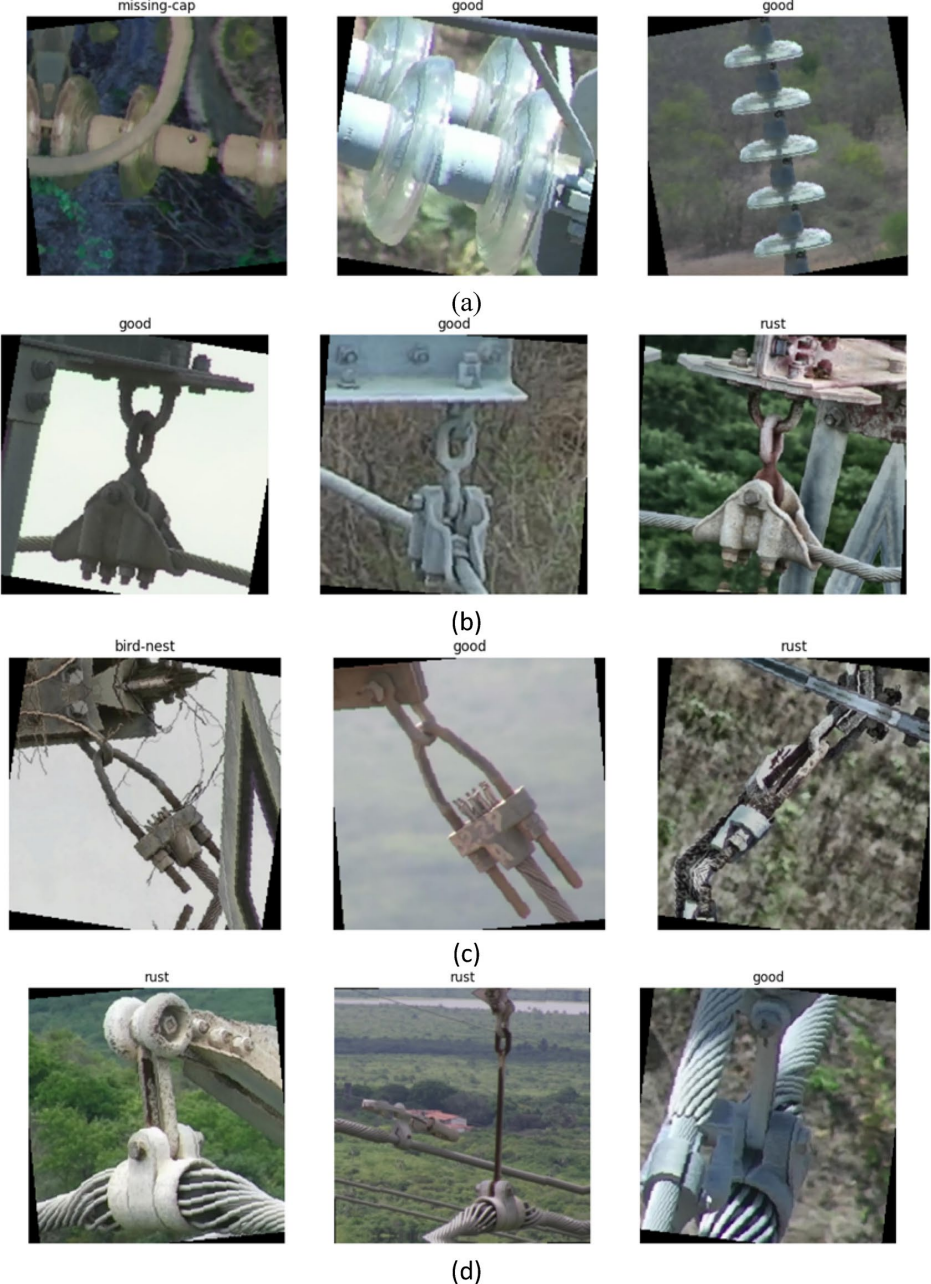
Samples of the InsPLAD dataset: (**a**) Samples of galss insulator, (**b**) Samples of lightning-rod-suspension insulator, (**c**) Samples of vari-grip insulator, (**d**) Samples of yoke-suspensioninsulator.

**Fig. 2:**
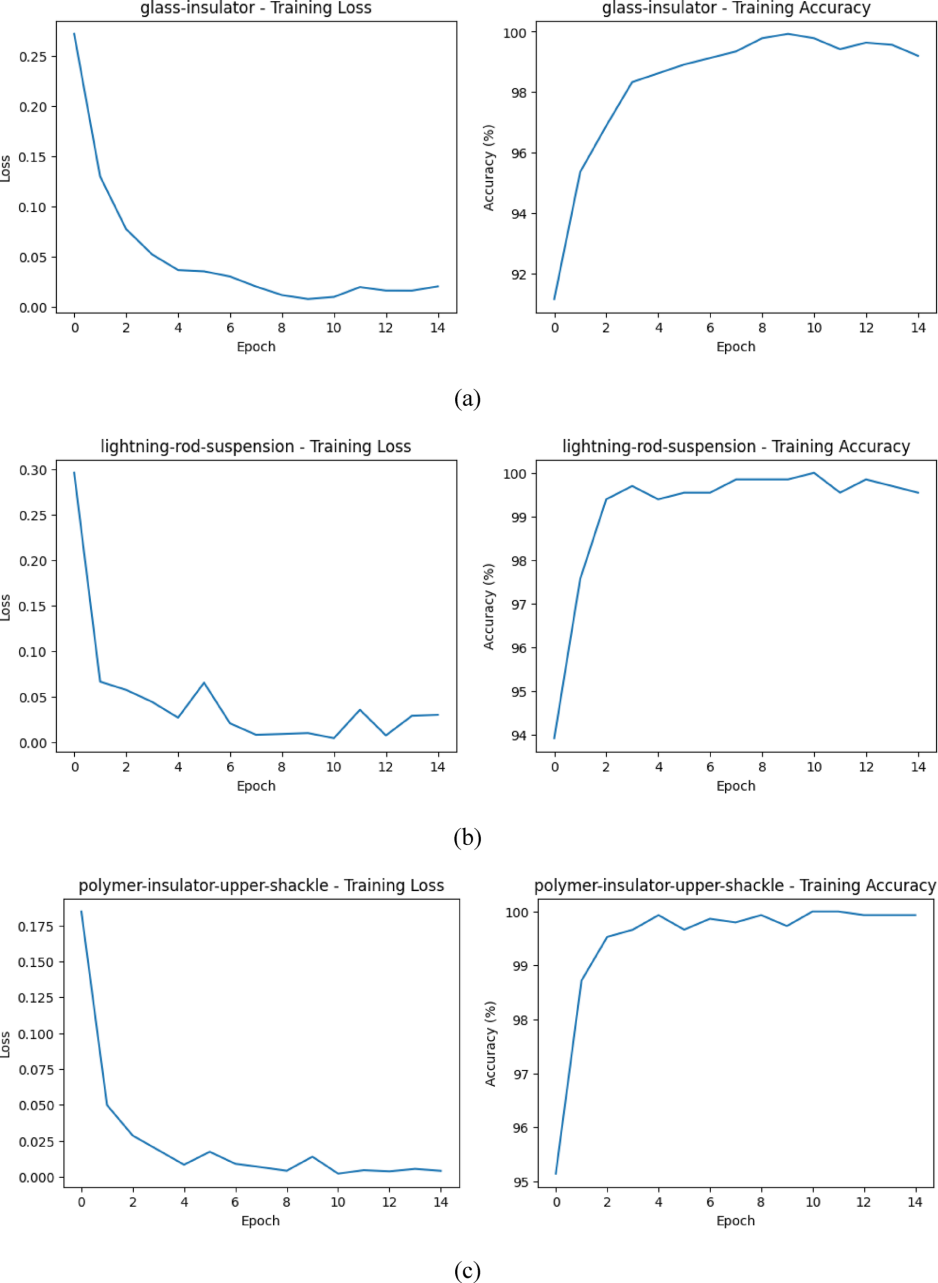

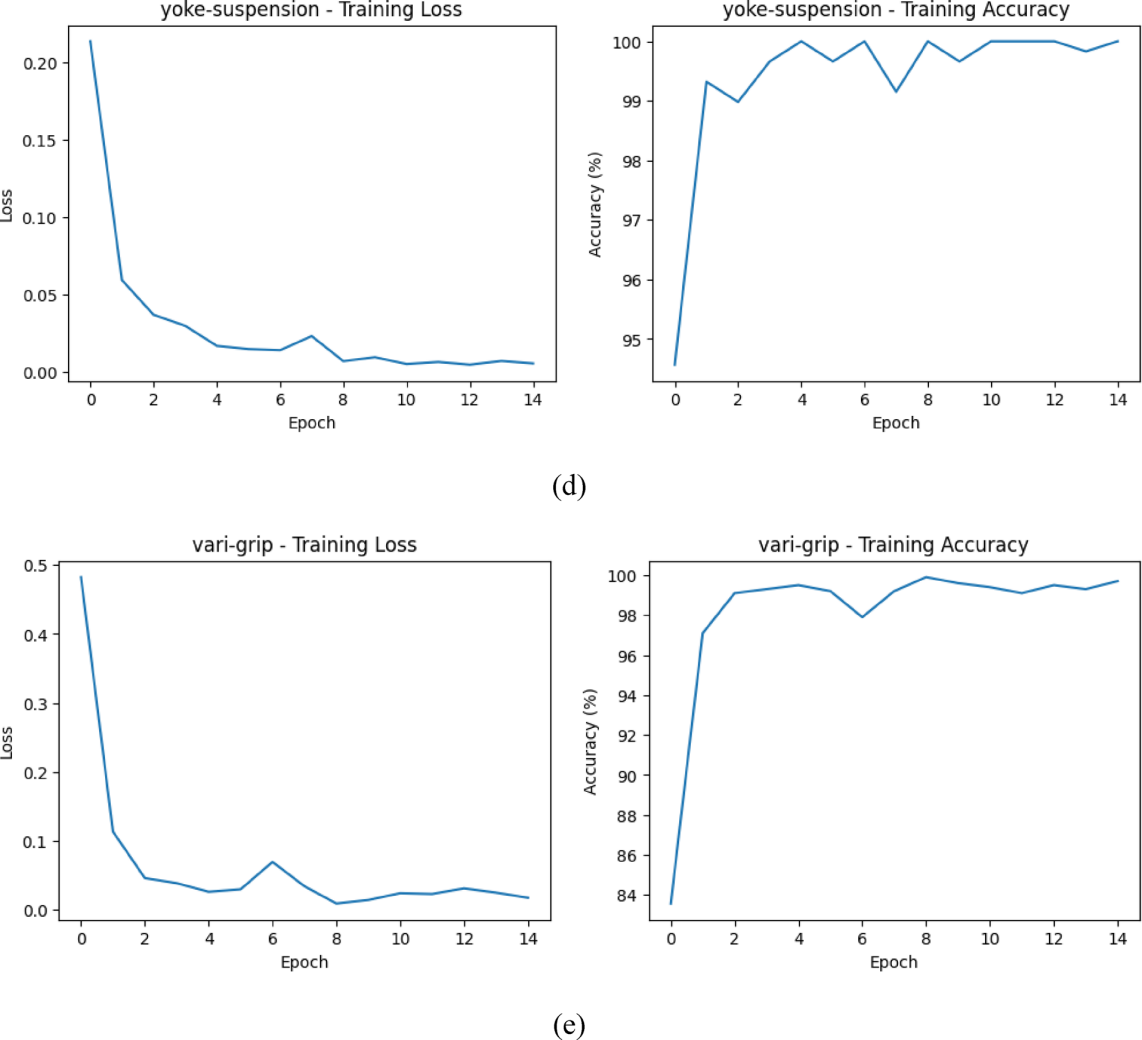
Training loss and accuracy for all classes.

**Fig. 3:**
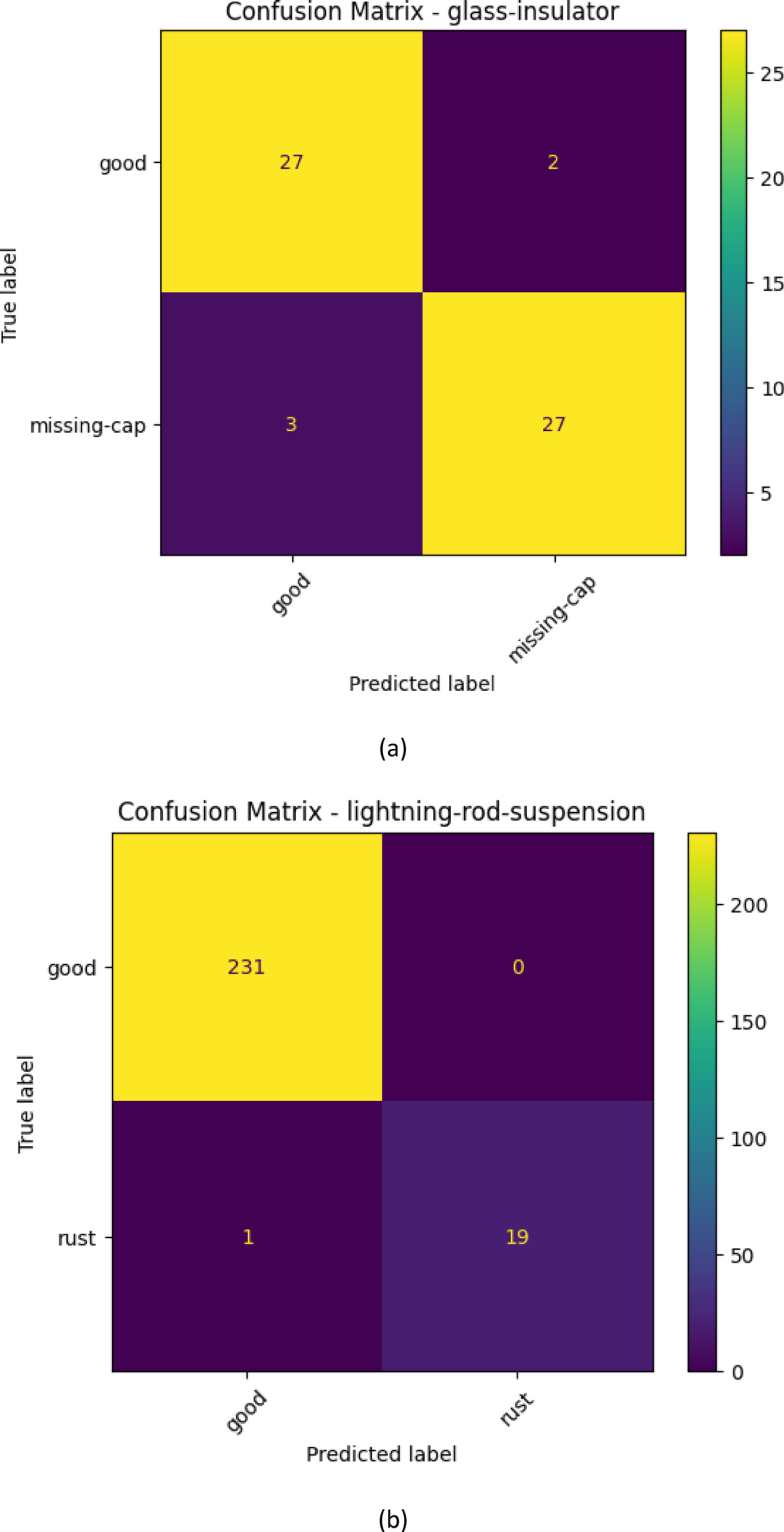

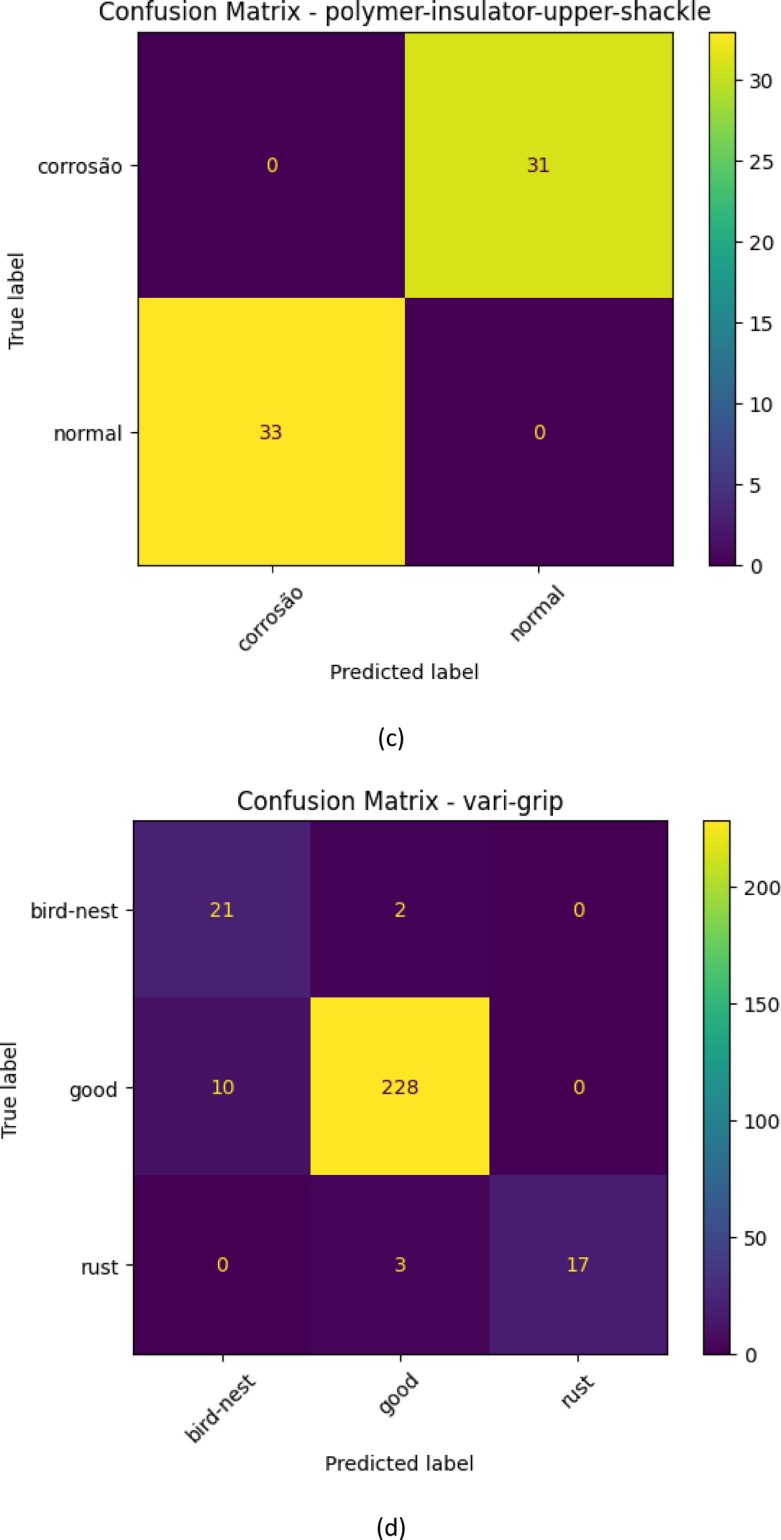

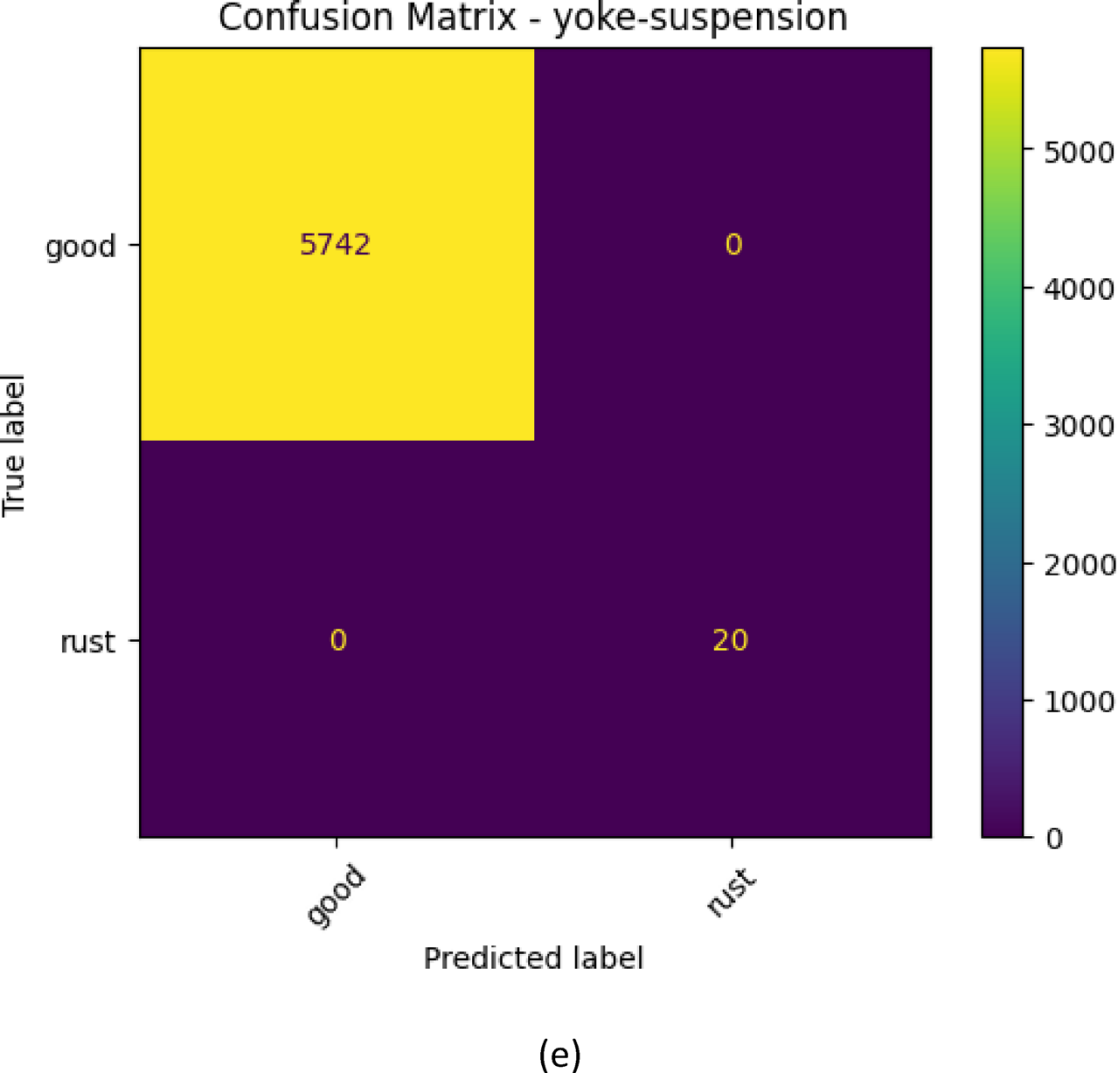
The confusion matrix for all classes.

In the correct operation of the OHTLs, it is possible to maintain a constant power supply to end users. There are conventional methods of testing the condition of high-voltage (HV) insulators, such as megger testing^2^, tap testing^3^, and on-foot visual inspection^4^. Foot visual assessment is the most ancient and most basic. To detect flaws and other problems that may destroy the power transmission system, an HV inspection engineer will physically examine and inspect the OHTLs. Although the megger test is performed with the help of the insulation resistance measure, the tap test is performed when an insulator is tapped to reveal weak connections. HV insulators are highly contaminated by their vulnerability and constant exposure to harsh climatic conditions. Therefore, leakage current occurs due to the conduction layer formed by the mixture of dust, mud, snow, and other pollutants (see Figs. 1, 2 and 3) and this enhances the chances of power interruptions^5,6^. The problem of contaminated HV insulators in OHTLs has been known over several years. This is the reason why governments and power utilities need to check on the status of HV insulator periodically and examine it.

The conventional techniques of inspecting high-voltage insulators are visual checks^7,8^, leakage current checks^9^, partial discharge checks^10^, and voltage polarity checks^11,12^. Visual inspections imply the visual examination of the state of the insulator with the help of unmanned aerial vehicle (UAV) devices^8^, Infrared (IR) thermography, or Ultraviolet Radiation (UR) cameras^7^. Leakage through the insulator surface is also measured when measuring leakage current. Partial discharge measurements are used to measure electrical discharge. To detect the patterns of aging and breakdown, Halloum et al.^11^ and Ogbonna et al.^12^ examine composite and polymeric outdoor insulators in high voltage direct current converter stations according to the variation in polarity of voltage. The voltage polarity reversal can cause degradation which can cause leakage current to increase^12^.

Deep learning (DL) and other advanced approaches are becoming more and more popular in power engineering. By using cutting-edge techniques, the shortcomings of traditional approaches may be monitored and the precision of the HV insulator status monitoring may be enhanced. Up to this date, HV insulator condition monitoring has been widely researched^13,14^. The classification of HV insulator surface contamination is based on recurrent neural networks (RNNs)^14^ and convolutional neural networks (CNNs)^13^.Owing to the ability of CNNs to learn and extract image features successfully, the networks are extremely useful in the classification of images^15^.

Recent advances in intelligent fault diagnosis have leveraged deep learning and Transformer-based architectures to improve feature extraction and classification performance. For instance, the study in^16^ introduces a multi-channel Transformer model with shift-window mechanisms for bearing fault diagnosis, effectively capturing relationships between segmented signal patches and enabling cross-domain adaptation. While this approach demonstrates strong performance in rotating machinery applications, it is primarily designed for single-task diagnosis and signal-based data, limiting its applicability to complex visual inspection problems such as HV insulator defect analysis. Moreover, it does not address the challenges of multi-task learning or the need for adaptive feature modulation across different defect categories.

Similarly, the work presented in^17^ proposes the LESSECT method, an advanced time–frequency analysis technique that improves instantaneous frequency estimation through entropy-based chirplet transform optimization. Although this method enhances the resolution and interpretability of non-stationary signals, its focus remains on signal processing rather than feature learning frameworks. As a result, it lacks integration with deep learning models that can perform end-to-end defect classification, and it does not consider multi-task scenarios or the extraction of hierarchical visual features required for image-based inspection systems.

In^18^, a multi-branch feature cross-fusion Transformer model (MCFormer) is introduced to address multi-sensor data fusion challenges in bearing fault diagnosis. This approach effectively combines local and global features through a structured fusion strategy, improving classification accuracy. However, despite its strong performance, the model does not explicitly address task-specific feature disentanglement, which may lead to negative transfer when extended to multi-task settings. Additionally, the framework focuses on multi-sensor data fusion within a single diagnostic objective, rather than handling multiple related tasks simultaneously or incorporating dynamic, case-aware feature adaptation mechanisms.

That class imbalance remains a fundamental challenge in fault diagnosis and defect detection systems, particularly under real-world operating conditions in discussed in many recent works. For instance, the work in^19^ demonstrates that severe class imbalance, combined with domain variability and limited training diversity, significantly degrades model generalization, and proposes an uncertainty-aware mixup and contrastive framework to enhance minority-class representation. Similarly^20^, addresses the issue of imbalanced data under unknown operating conditions by introducing a prototype-guided supervised contrastive learning strategy with dynamic temperature modulation and class-balanced loss, enabling improved learning of domain-invariant features while reducing bias toward dominant classes. Furthermore^21^, investigates long-tailed data distributions in multi-domain settings and proposes a two-stage learning framework that combines domain-invariant feature alignment with balanced classifier learning, effectively mitigating overfitting to majority classes. Despite these advancements, most existing approaches are primarily designed for signal-based rotating machinery diagnosis and rely on specialized balancing strategies within single-task frameworks, limiting their applicability to complex multi-task visual inspection problems such as HV insulator defect classification. In addition, these methods often lack mechanisms for multi-scale feature modeling and dynamic task-aware adaptation, which are essential for handling heterogeneous defect patterns in power transmission systems.

Recent advances in contrastive and self-supervised learning have significantly improved model generalization under challenging industrial conditions, particularly in scenarios involving domain shifts, limited labeled data, and unknown fault types. For example, the study in^22^ proposes an open-set domain generalization framework that integrates data generation and adversarial feature learning to simultaneously address class gaps and domain gaps, enabling robust recognition of both known and unknown fault categories. Similarly^23^, introduces a physics-informed semi-supervised domain generalization framework that combines contrastive learning with physical consistency constraints, allowing the model to learn domain-invariant representations while effectively leveraging unlabeled data. These approaches highlight the growing importance of contrastive representation learning, domain generalization, and hybrid learning strategies in enhancing robustness and adaptability. However, despite these advancements, most existing methods are tailored to signal-based fault diagnosis in rotating machinery and focus on single-task learning settings, with limited exploration of multi-task visual inspection problems. Moreover, they often lack mechanisms for multi-scale feature extraction and dynamic case-aware adaptation, which are critical for handling heterogeneous defect patterns in HV insulator inspection.

There are however two large disadvantages to single task methods. To start with, deploying and computing the costs are increased when separate models are trained on individual types of components. Second, these models do not give good overall generalization due to their failure to utilize common visual characteristics across different defects situations, including corrosion marks, texture flaws, and edge discontinuities. Multi-task learning (MTL) frameworks have been created to address these limitations. These frameworks keep task-specific output layers and have a common backbone between a few related tasks. Through inductive transfer between tasks, hard parameter sharing, in which all tasks share the identical feature extractor has been shown to promote generalization and reduce overfitting.

In an attempt to overcome the above challenges, the paper suggests a Multi-Scale Case-Aware Dynamic Feature Modulation framework with Self-Supervised Pretraining (MS-CADFM-SSL) to multi-task defect classification. In the suggested approach, an EfficientNet backbone that has been trained based on contrastive self-supervised learning is used to obtain strong defect-invariant representations. Case-aware attention mechanisms are used to extract multi-scale features and dynamical modulate them to use the model to highlight task-relevant characteristics in various semantic levels. Also, to decrease feature redundancy and negative transfer, an orthogonality regularization method is used to induce variety among task-specific modulators explicitly. The proposed framework is perpetually and significantly better than traditional single-task and multi-task baselines, which is demonstrated by numerous tests and ablation studies. It also provides superior interpretability with the help of feature similarity analysis and visual explanations.

The main contributions of this work are summarized as follows:

- A novel case-aware dynamic feature modulation mechanism for adaptive multi-task defect classification.
- A multi-scale task-aware architecture that captures complementary defect characteristics across different feature levels.
- An orthogonality-regularized learning strategy to enhance task specialization and reduce negative transfer.
- The integration of self-supervised pretraining to improve performance under limited labeled data conditions.

The rest of the parts of the paper will be organized as follows: Sect.  2 will discuss the dataset and the preprocessing techniques. Section  3 illustrates the suggested model. Results of the experiment will be discussed in Sect.  4 and the conclusion will be in Sect.  5.

## Dataset

### Datasplit

The Insulator Power Line Asset Dataset (InsPLAD) Dataset^24^ is directly dedicated to the identification of power insulators and the related elements. The resolution of the image is mostly 512 × 512 dots, with a considerable amount of combined samples of insulators. The dataset that was used in this study is composed of InsPLAD dataset of power transmission components that is divided into five different fault cases, including glass insulators, lightning rod suspensions, polymer insulator upper shackles, vari-grip components, and yoke suspensions. Each of the different types of failures that occur in the real world power infrastructure is reflected in every scenario, and it represents a specific supervised classification problem with its own fault categories. The split of the dataset is a standardized train validation split in order to support reliable model building and assessment. An overview of the data is tabulated in Table 1.

The data indicate there was a significant variation in the total sample and the distribution of classes in the five cases. The training examples provided by certain situations, such as glass-insulator and polymer-insulator-upper-shackle, have a relatively balanced normal and faulty training data. Nevertheless, in more cases, the balance of classes is not even, and, particularly, in the validation sets, certain defect classes are heavily underrepresented. This difference is a mirror image of the real situation of inspections when defects are not very common compared to normal operation conditions. It is worth noting that the yoke-suspension case shows severe lack of balance in the validation set where there is a high population of normal samples in comparison to a small population of defective samples. Equally, vari-grip and lightning-rod-suspension cases exhibit disproportional distributions of types of defects. These features render the dataset especially problematic and highly suitable to testing sophisticated multiple-task learning methods, dealing with class imbalance solutions, and generalization ability in the real-life environment. In Fig. 1, samples of the dataset are presented.

### Processing and data augmentation

The information is received because of the integration of the mobile camera shots and images obtained through the Internet. Image resolution, angle and consistency problems are problematic with this compromise character. Consequently, it is necessary to have a preprocessing stage so as to control this issue. In this section, the list of the preprocessing techniques will be listed. The normalization has the benefit of speeding up convergence of training and reducing the effect of varying image intensities. Standardization or (Z-Score Normalization ) transfigures data such that it has a mean of 0 and a unit variance as shown in Eq. 1.

1 $$\:{I}_{normalized}=\:\frac{I-\:\mu\:}{\sigma\:}$$

Where:

$\:I$ : is the original image’s pixel intensity.

σ: is the standard deviation of the pixel intensities.

µ: is the mean of the pixel intensities.

To address the severe class imbalance in the dataset, we applied several data augmentation techniques aimed at increasing the diversity and robustness of the training data. These augmentations include both geometric transformations and color-based alterations to simulate different environmental conditions. Specifically, random rotations, translations, scalings, shears, flips (left-right and up-down), and cropping were employed to replicate various perspectives of the electronic components. These transformations help the model generalize better across different orientations and shapes of defects in power line components, especially in cases with limited samples for certain defect types.

In addition to geometric changes, color-based augmentations were implemented to simulate variations in lighting conditions, which are common in real-world inspection scenarios. These include random adjustments to brightness, contrast, saturation, and hue, allowing the model to become more robust to changes in environmental lighting. By introducing such variations, the model is better equipped to handle the natural variability in images captured under different conditions, thus improving its performance on imbalanced datasets.

To ensure that the augmentations do not bias the model, new hyperparameter ratios for each augmentation technique were applied at every training epoch. This dynamic adjustment enables the model to experience a wide range of transformations throughout the training process, further increasing the diversity of the data and helping to mitigate the risk of overfitting to any specific augmentation pattern.

**Table 1:** InsPLAD Database summary.

Case	Split	Class	Samples	Total per split
Glass Insulator	Train	Good	691	1381
		Missing-Cap	690	
	Val	Good	29	59
		Missing-Cap	30	
Lightning Rod Suspension	Train	Good	348	658
		Rust	310	
	Val	Good	231	251
		Rust	20	
Polymer Insulator Upper Shackle	Train	Corrosion	740	1482
		Normal	742	
	Val	Good	31	64
		Rust	33	
Vari-Grip	Train	Bird-Nest	350	998
		Good	358	
		Rust	290	
	Val	Bird-Nest	23	281
		Good	238	
		Rust	20	
Yoke Suspension	Train	Good	299	589
		Rust	290	
	Val	Good	5742	5762
		Rust	20	

## The proposed pipeline

### Problem definition and dataset context

Where D = {D1, D2, ……, DT } is a set of defect cases, and each defect case Dt relates to a supervised image classification task of its own label space, independent, that is, of the model. In this work, T = 5, which is a total of five power transmission component categories namely; glass insulators, lightning rod suspensions, polymer insulator upper shackles, vari-grip components and yoke suspensions. A separate training and validation split in each of the cases is provided, with different numbers of defect classes, and considerable variances in the class distribution.

Even though these defect cases have some common visual features, including structural edges, surface textures, corrosion patterns and material discontinuities, each case highlights different discriminative cues based on the component geometry and the failure mode. To illustrate, the absence of caps in glass insulators would have completely different fundamentals of corrosion patterns found on metallic suspension parts. Such heterogeneity causes naïve parameter sharing to be suboptimal and encourages a learning strategy that makes a tradeoff between shared representation learning on one hand, and task-specific adaptation on the other.

The aim of the research paper is thus to study a single model that (i) shares visual knowledge among cases of defects, (ii) shapes features according to the case, and (iii) is robust even in the case of a very strong imbalance of classes and a very limited amount of labelled data, as presented in the given dataset.

### Self-supervised pretraining on unlabeled defect images

In order to deal with the issue of scarcity of labeled defect samples and enhance the robustness of representations, the backbone network is initially pretrained on a contrastive self-supervised learning approach relying on SimCLR^25^. In this phase, class annotations are not exploited to make use of unlabeled images of all defect cases.

With two randomly augmented views xi and xj of the same input image, the network is trained to achieve consensus between the latent representation of xi and xj and to be dissimilar to the latent representations of other images in the batch. This is done by a contrastive loss that is calculated in the embedding space. Through the application of self-supervised pretraining, the backbone is able to learn defect-invariant visual primitives including edges, surface roughness, corrosion textures and shape irregularities. Such characteristics are of particular significance to inspection of power infrastructures with flaws that tend to have low-level surface visual correspondence features in the various components. The already trained backbone is then refined in the course of supervised multi-task learning.

### Multi-Scale feature extraction with EfficientNet

Multi-Scale feature extraction with EfficientNet aims to improve the use of multiple scales to capture essential details about the image.<|human|> Multi-Scale feature extraction with EfficientNet. The approach will enhance the application of multiple scales to extract crucial information about the image. The framework suggested is based on EfficientNet^26^ as a common core because of its good performance-efficiency trade-off strategy and compound scaling policy. EfficientNet generate hierarchical feature representations on various depths, which allows visual information to be captured on various semantic levels.

Where f1,f2,…,fS are the extracted feature vectors of S sampled backbone stages feature vectors where lower-level features represent fine-grained textures and edges, and higher-level features represent semantic and structural information. Such a multi-scale representation is especially useful to the classification of defects, where some defects can occur at localized scales (e.g., corrosion), but others are characterized by global structural anomalies (e.g., missing components).

### Multi-scale case-aware dynamic feature modulation (MS-CADFM)

In order to accommodate shared representations to the non homogeneous defect cases, there is a Multi-Scale Case-Aware Dynamic Feature Modulation (MS-CADFM) mechanism. In every defect instance t and feature scale S, a specific modulation function is trained to learn a relevance to task-reweight the aspects of the backbone features. Formally, the modulated feature at scale s for task t is defined as shown in Eq. 2:

$$\:{\stackrel{\sim}{f}}_{t}^{s}=\:{\alpha\:}_{t}^{s}\:\:\odot\:\:{f}^{s}\:\:,$$

2 $$\:where\:\:\:\:{\alpha\:}_{t}^{s}=\:\sigma\:\:\left(\:{W}_{t}^{s}{f}^{s}\:\right)\:$$

$\:{W}_{t}^{s}\:$represents parameters of the modulation network that can be learned, σ( ) is a sigmoid activation, and denotes element-wise multiplication. The operation allows the model to highlight the channels that are relevant to the task and inhibit the irrelevant features.

The multi-scale modulated features will then be concatenated to create a task aware representation as illustrated in Eq. 3:

3 $$\:{\stackrel{\sim}{f}}_{t}^{s}=\left[{\stackrel{\sim}{f}}_{t}^{1\:\:}\left|\right|{\:\stackrel{\sim}{f}}_{t}^{2\:\:}\dots\:\dots\:\dots\:\dots\:.\left|\right|{\:\stackrel{\sim}{f}}_{t}^{S\:\:}\right]$$

The design enables the model to dynamically scale shared knowledge to every case of defects without redundancy of the underpinning network.

### Orthogonality regularization between case modulators

Although case-aware modulation contributes to greater adaptability, the case-aware modulators can cause negative transfer when there is too much similarity between tasks. In order to directly promote diversity and specialization, an orthogonality regularization term is added between the modulation matrices of complementary cases of defects.

The loss of orthogonality is characterized as below in Eq. 4:

4 $$\:{l}_{ortho}=\:\sum\:_{t\ne\:k}{{\left|\right|M}_{t}^{T}\:{M}_{k}\left|\right|}_{F}^{2}$$

where $\:{M}_{t}$ and $\:{M}_{k}$​ denote the modulation parameter matrices for tasks $\:t\:$and $\:k$, respectively, and $\:{\parallel\:\cdot\:\parallel\:}_{F}$ is the Frobenius norm. This regularization enforces decorrelated attention patterns across cases, promoting task-specific discrimination and reducing feature redundancy.

### Optimization objective

The overall training objective combines task-specific classification loss with orthogonality regularization as shown in Eq. 5:

5 $$\:{l}_{total}=\:\sum\:_{t=1}^{T}{L}_{CE}^{t}+\lambda\:{l}_{ortho}$$

where $\:{L}_{CE}^{t}$​ denotes the class-weighted cross-entropy loss for task ttt, designed to mitigate severe class imbalance observed in the dataset. The hyperparameter λ controls the contribution of the orthogonality constraint.

### Pseudo-code for the MS-CADFM

The proposed pipeline begins by addressing the scarcity of labeled defect data through self-supervised pretraining using a contrastive learning strategy (SimCLR). In this stage, the model learns general visual representations from unlabeled images by maximizing agreement between different augmented views of the same image while distinguishing them from others. These learned representations capture defect-invariant features such as edges, textures, and corrosion patterns. The pretrained backbone, based on EfficientNet, is then used to extract multi-scale feature representations, where lower-level layers focus on fine-grained details and higher-level layers capture semantic and structural information. This multi-scale design ensures that both localized defects and global anomalies are effectively represented.

To handle the heterogeneity across different defect cases, the framework introduces a Multi-Scale Case-Aware Dynamic Feature Modulation (MS-CADFM) mechanism. This module adaptively reweights feature channels for each task using learned modulation parameters, enabling the model to emphasize task-relevant features while suppressing irrelevant ones. The modulated features from multiple scales are concatenated to form a task-specific representation, which is then fed into dedicated classification heads. To further improve task specialization and prevent negative transfer, an orthogonality regularization term is applied between task-specific modulation parameters, encouraging diversity across tasks. The overall model is trained end-to-end using a combination of class-weighted cross-entropy loss (to address class imbalance) and the orthogonality constraint, resulting in a robust and adaptive multi-task learning framework. The Pseudo-code for the MS-CADFM is listed below:

**Algorithm 1:**
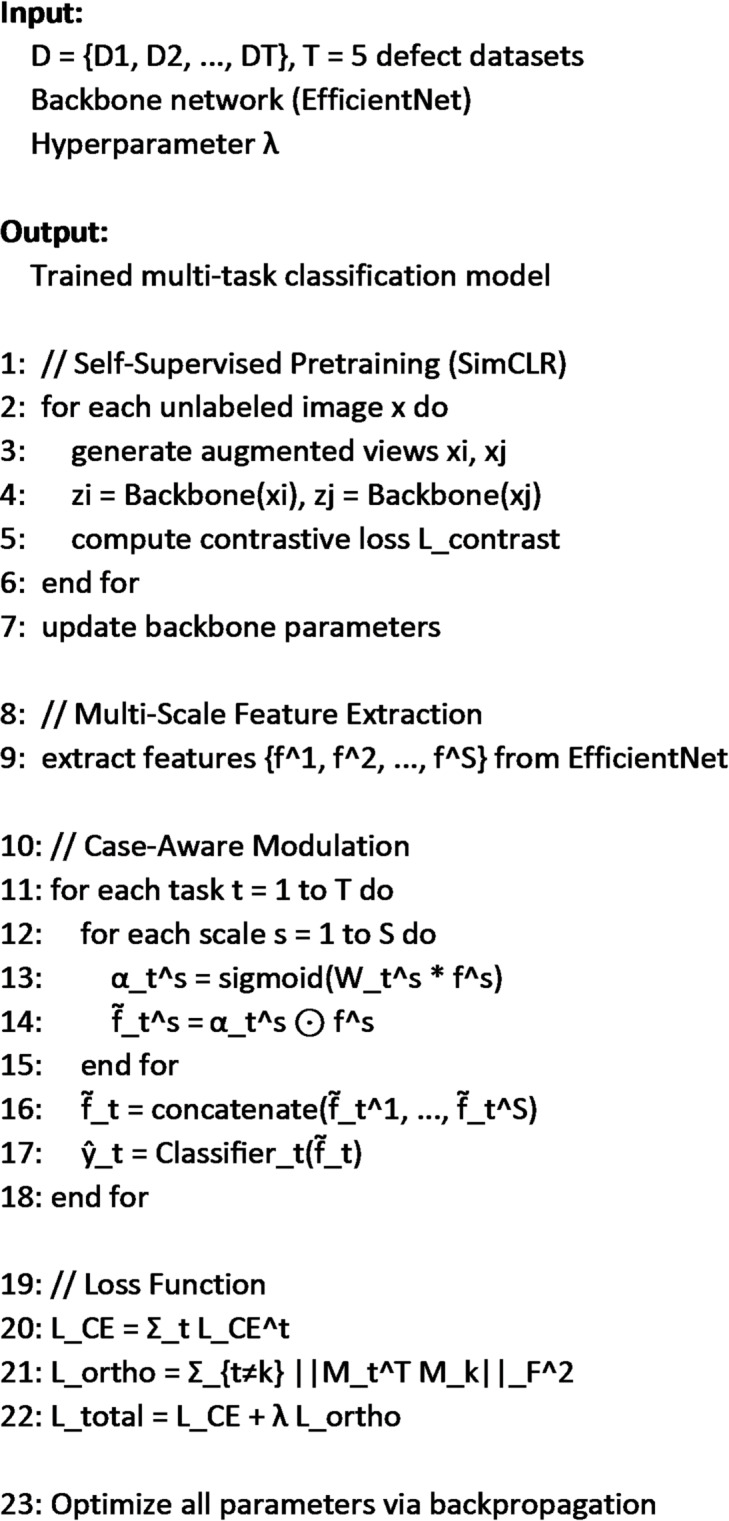
MS-CADFM defect classification pipeline.

## Simulation analysis and results

### Evaluation metric

The recall and precision are the performance indicators of the models. Precision is defined in Eq. (6) as the ratio of the number of true positive samples or the samples that had been correctly taken to be objects and the total number of positive samples. The fraction of true positive samples: the correctly identified samples that was identified as an object to the sample that was identified correctly, is referred to as the recall, represented in Eq. (7). The F1 Score uses the algorithm to optimize these measurements as shown in Eq. (8). False positives, false negatives, and true positives have an impact on the F1 Score. An upsurge in the false negatives would reduce the recall and an upsurge in false positives will reduce the precision resulting in a smaller F1 Score. On the other hand, the true positives are increased which results in better memory and precision and an increase in the F1 Score.

6 $$\:\mathrm{P}\mathrm{r}\mathrm{e}\mathrm{c}\mathrm{i}\mathrm{s}\mathrm{o}\mathrm{n}\:=\:\frac{TP}{TP+FP}$$

7 $$\:\mathrm{R}\mathrm{e}\mathrm{c}\mathrm{a}\mathrm{l}\mathrm{l}\:=\:\frac{TP}{TP+FN}$$

8 $$\:F1=\frac{2*precision*recall}{precision+recall}$$

## Specification for experiments

The experiments of the study are carried out on Windows 11 on the Colab T4 GPU with 15GB VRAM. The deep learning engine employed in the creation is PyTorch 1.10. The Adam optimizer was used during the training process with the learning rate of 0.0001. The networks of the tests are all trained without using the official pre-training weights to give a fair comparison of the 15 epochs.

To ensure reproducibility and provide a complete description of the proposed architecture, we further specify the key implementation details and hyperparameters used in the MS-CADFM-SSL framework. The EfficientNet backbone is initialized without ImageNet pretraining in order to fairly evaluate the effect of self-supervised learning, and feature maps are extracted from multiple stages corresponding to different spatial resolutions (S = 3 scales). Each modulation module $\:{W}_{t}^{s}$ is implemented as a lightweight fully connected layer followed by a sigmoid activation, and the channel-wise feature dimension is kept consistent across scales to enable effective concatenation in Eq. (3).

During training, the model is optimized using the Adam optimizer with an initial learning rate of 1 × 10^− 41^, β₁ = 0.9 and β₂ = 0.999, and a batch size of 32. The number of training epochs is set to 15 for all experiments to ensure fair comparison across baselines and to prevent overfitting on relatively small datasets. It is important to note that the backbone benefits from the prior self-supervised pretraining stage, which provides strong initialization and reduces the need for prolonged supervised training. The self-supervised pretraining stage uses a SimCLR-style contrastive loss with temperature τ = 0.5. For the multi-task learning phase, the orthogonality regularization coefficient λ in Eq. (5) is empirically set to 0.1 unless otherwise stated in the sensitivity analysis. Standard image resolution is fixed at 224 × 224 pixels after preprocessing and augmentation. These settings are kept consistent across all five defect cases to ensure stable and unbiased evaluation of the proposed framework.

### Experimental results

Table 2 gives the overall performance of the classification in five components and it can be seen that the proposed model has consistently good results in general. In the case of Glass-Insulator and Lightning-Rod-Suspension, the values of the precision are very high (93.1% and 100%), which means that when the model predicts such classes it is normally accurate. Lightning-Rod-Suspension achieve (95.0) recall and, accuracy of (91.9). Conversely, Glass-Insulator is more balanced when it comes to accuracy and recall and this gives an excellent F1-score of 91.53.

Polymer-Insulator-Upper-Shackle and Vari-grip are the two polymer materials that the model performs very well with. Precision and recall in both classes are near-perfect, resulting in F1-scores of very close to 1.0 and very high values of accuracy (99.9% and 99.8%). The findings imply that these components have visual characteristics that are very discriminative and the model can easily differentiate them with other classes with minimum confusion. The small difference between the precision and the recall of Vari-grip does indicate that a few samples are being misclassified, though it does not have a major impact on the overall performance. In the case of yoke-suspension, the model is almost perfectly behaved, with accuracy and recall statistics of about 0.99 and F1-score of 0.99 respectively. This means that there are very low false positives and false negatives which proves that the classifier is robust when dealing with this component. On the whole, Table 2 indicates that the model is very effective in most classes, and the performance differences can be mostly explained by the bias in classes and the distribution of data, as opposed to the constraints of the model. The training loss and acuuracy for each class is shown in Fig. 2.

**Table 2:** The Classification report.

Class	Precision	Recall	F1-score	Accuracy
Glass-Insulator	93.1	90.0	91.53	91.67
Lightning-Rod-Suspension	94.2	95.2	97.5	91.9
Polymer-Insulator-Upper-Shackle	100	100	99.99	99.9
Vari-grip	98.52	90.73	98.81	99.8
yoke-suspension	99.0	0.99	0.99	99.9

The glass-insulator case demonstrates that the performance of the classification is good and balanced since the confusion matrix illustrates. Both normal and missing-cap classes are properly identified and misclassifications are few. The high sensitivity and the recall percentages suggest that the proposed MS-CADFM-SSL system is effective in capturing the structural characteristics that aid in distinguishing between intact insulators and missing-cap defects although it has a small validation sample.

With the lightning-rod-suspension, the model is virtually ideal in making distinctions of good and rusted parts. Based on the confusion matrix, the normal samples have been perfectly classified and only one rust has not been detected. This observation highlights the effectiveness of the given method when the imbalance between the classes is severe and demonstrates the practicality of the class-weighted loss and case-aware modulation in the capability to retain the sensitivity to the infrequent defect patterns.

An example of such a case is the polymer-insulator-upper-shackle case because it is highly unlikely to differentiate between a corrosion and a normal case due to high visual correlation. According to the confusion table, the model is more concerned with detecting defects and hence the capacity to label all the corrosion samples appropriately with a flawless recall. This increases the error rate, but in the instance of safety critical inspection, this is better than a malfunctioning part getting through undetected and that is far costlier than false alarms.

The vari-grip case is mostly showing that the model is doing well with minimal misclassifications. The good class is dominant having the right number of 228 out of 238 samples of the category which is correctly identified and hence high precision in the category. The two groups of bird-nest and rust are, however, somewhat confused: 10 of the good group were determined as bird-nest, and 3 as rust. This means that even though the model is quite aggressive in their presentation of the predictions of what would be deemed as good, it may not be very effective in distinguishing the defective types in some instances particularly where the defective traits are so small or related in appearance with other groups.

This is so well executed and the model is almost perfect in the case of yoke-suspension. The 5742 good samples were correctly classified and 20 rust samples too were predicted. There is no indication of misclassification and we may suppose that the properties defining the good and rust are highly discriminative and can be easily described through the model. A difference between vari-grip confusion matrix this large demonstrates that the complexity of classification and inter-class similarity have significant effect on the model performance with simpler distinctions giving the model a near perfect accuracy of classification and more complex defects giving the model a few errors. The confusion matrix for each case is shown in Fig. 3.

**Table 3:** comparative performance with state-of-the-art methods.

Method	Precision (%)	Recall (%)	F1-score (%)	Accuracy (%)
MCFormer^21^	94.8	93.2	94.0	94.5
OISDG^22^	95.6	91.0	93.2	94.1
DTM-PSCL^23^	96.2	92.8	94.5	95.0
OSDG^25^	94.1	90.5	92.2	93.6
MS-CADFM (no SSL) (ours)	95.3	93.6	94.4	95.1
MS-CADFM + SSL (ours)	97.1	95.2	96.1	96.8
**MS-CADFM-SSL (Full) (ours)**	**98.5**	**96.9**	**97.7**	**98.2**

The comparative results in Table 3 indicate that recent advanced methods such as MCFormer^18^ and DTM-PSCL^20^ achieve strong performance due to their powerful feature extraction and representation learning strategies. Specifically, MCFormer attains 94.8% precision, 93.2% recall, and 94.0% F1-score, demonstrating its effectiveness in capturing discriminative features through multi-branch fusion. Similarly, DTM-PSCL achieves 96.2% precision and 94.5% F1-score, benefiting from its contrastive learning mechanism that enhances class separability. These results confirm that both approaches are highly competitive baselines, particularly in structured fault diagnosis scenarios where data distributions are relatively stable and tasks are homogeneous.

However, despite these strong results, their performance remains slightly lower than the proposed method, particularly in terms of recall and overall accuracy. For example, while DTM-PSCL achieves 92.8% recall and 95.0% accuracy, and MCFormer achieves 93.2% recall and 94.5% accuracy, these values are still below the performance of the proposed framework. This gap highlights a key limitation: these methods are primarily designed for single-task or signal-based diagnosis, and therefore lack explicit mechanisms for handling multi-task heterogeneity and visual variability across different defect categories. As a result, their ability to generalize across diverse defect cases is constrained, especially under imbalanced conditions.

In contrast, the proposed MS-CADFM-SSL framework consistently achieves the best performance across all evaluation metrics, reaching 98.5% precision, 96.9% recall, 97.7% F1-score, and 98.2% accuracy. Even intermediate variants of the model demonstrate clear improvements. For instance, the MS-CADFM model without self-supervised pretraining achieves 95.3% precision and 94.4% F1-score, already outperforming most baselines. When self-supervised learning is introduced, performance increases further to 97.1% precision and 96.1% F1-score, highlighting the effectiveness of representation pretraining. These incremental improvements clearly show that the gains are not incidental, but arise from the progressive integration of the proposed components.

Furthermore, the ablation study in Table 4 provides deeper insight into the contribution of each module. The baseline EfficientNet achieves only 86.9% F1-score and 87.2% accuracy, while adding self-supervised learning alone improves performance to 91.2% F1-score. Introducing case-aware modulation further boosts performance to 92.6% F1-score, confirming its role in enhancing task-specific feature discrimination. The combination of both (SSL + CADFM) leads to a significant jump to 96.1% F1-score, demonstrating strong synergy between representation learning and task adaptation. Finally, the full model with orthogonality regularization reaches 97.7% F1-score and 98.2% accuracy, confirming that orthogonality constraints effectively reduce negative transfer and refine feature specialization. These results collectively validate that each component contributes meaningfully, and that the overall framework operates as a coherent and synergistic system rather than a simple combination of existing techniques.

**Table 4:** Ablation study of proposed components.

Model Variant	SSL	CADFM	ORTHO	Precision (%)	Recall (%)	F1 (%)	Accuracy (%)
Baseline (EfficientNet)	✗	✗	✗	88.3	85.7	86.9	87.2
+ SSL only	✓	✗	✗	92.4	90.1	91.2	91.8
+ CADFM only	✗	✓	✗	93.8	91.5	92.6	93.1
+ SSL + CADFM	✓	✓	✗	97.1	95.2	96.1	96.8
Full Model	✓	✓	✓	98.5	96.9	97.7	98.2

## Conclusions and future work

We have suggested a new MS-CADFM-SSL framework to classify defects in various power line components, using a common EfficientNet backbone, multi-scale case-aware dynamically modulated feature, Orthogonality regularization, and self-supervised pretraining, in this work. The model was tested on five cases of heterogeneous defects, and it showed good results with regards to precision, recall, F1-score, and accuracy. The framework can compromise between generalization and specialization in a multi-task defect inspection scenario by learning the shared representation where the discriminative information remains task specific.

Industrially, the suggested method has immense advantages to the power transmission as well as utility inspection activities. The frequency of recall of critical defect classes prevents the fact that safety risks, including the absence of insulator parts or corroded polymer shackles are easily identified and the number of operational failures is reduced. Moreover, the unified backbone lowers the cost of computation, allowing it to run on edge devices or real-time monitoring systems, which is needed when inspecting a large-scale system over a long transmission line.

MS-CADFM-SSL is better than traditional single-task models or naive multi-task approaches in terms of ability to adapt to imbalanced and visually diverse defects. Multi-scale feature modulation promotes interpretability as well as permits precise localization of defect areas, whereas the orthogonality regularization approach decreases negative transfer across tasks. Grad-CAM visualizations prove that the framework is not only the one that is capable of high classification performance but also concentrates on physically significant parts, which is relevant to automated reporting and human-in-the-loop validation in industry practice.

Even with these benefits, there still exist some constraints. Very small or infrequent defect patterns can still be wrongly classified even in classes with a very small number of validation samples. Moreover, the existing research is based on data of static images, which might not be reflecting the dynamic processes of the environment i.e. weathering, dust deposition, or light changes. These constraints indicate that there is a necessity of ongoing data collection and augmentation plans in order to enhance soundness in practical implementation conditions.

The extension of the framework to time and multi-modes as well as the incorporation of video footage, thermal imaging, and LiDAR based scans of the surface will be investigated in future studies in order to improve the reliability of defect detection. Also, the implementation of semi-supervised and active learning methods might help decrease the dependence on large labelled datasets and will make the method more scalable. Last but not least, the framework will be further developed to forecast the severity of defects and the priority of maintenance and this can be directly used in industrial decision making and predictive maintenance policies in power transmission networks.

## Data Availability

The dataset analysed during the current study are available on this link [https://github.com/andreluizbvs/InsPLAD](https:/github.com/andreluizbvs/InsPLAD).
